# Performance of the Dutch Triage standard in managing fever in children in out-of-hours primary care: a secondary analysis of the chili study

**DOI:** 10.1093/fampra/cmag015

**Published:** 2026-04-07

**Authors:** Robin N Gottwald, Eefje G P M de Bont, Jochen W L Cals, Laure Wynants

**Affiliations:** Department of Epidemiology, CAPHRI Care and Public Health Research Institute, Maastricht University, Maastricht 6229 HA, The Netherlands; Department of Family Medicine, Care and Public Health Research Institute (CAPHRI), Maastricht University, Maastricht 6229 HA, The Netherlands; Department of Family Medicine, Care and Public Health Research Institute (CAPHRI), Maastricht University, Maastricht 6229 HA, The Netherlands; Department of Epidemiology, CAPHRI Care and Public Health Research Institute, Maastricht University, Maastricht 6229 HA, The Netherlands; Department of Development and Regeneration, KU Leuven, Leuven 3000, Belgium

**Keywords:** triage, fever, pediatrics, after-hours care, clinical decision-Making, general practice

## Abstract

**Background and objectives:**

The Dutch Triage Standard (NTS) supports nurses in assessing case urgency in out-of-hours general practice (GP) care; however, its suitability for fever-related pediatric cases remains debated. This study examines the agreement between pre-consultation NTS urgency classifications (U-scores) and post-consultation management decisions in febrile children, and whether the NTS reduces between-center variation in decision-making.

**Methods:**

We performed a secondary analysis of the CHILI study, including 22 089 consultations for children under 12 with fever, across 18 out-of-hours GP centers (2015- 2016). Adjusted logistic regression models, with natural cubic splines and random intercepts, explored the associations between U-scores and three outcomes: referral to secondary care, antibiotic prescription, and other medication use. Intraclass correlation coefficients assessed between-center variability.

**Results:**

Unadjusted and adjusted percentages of the three outcomes showed similar trends. 24.5% of consultations were classified at low urgency levels (U4-5) before the consultation. Adjusted referral probabilities decreased with lower urgency: 29% (U1) to 0.8% (U5). Strikingly, antibiotic prescription rates were highest for U3 (26.2%) and U4 (25.1%). The probability of other medication prescriptions decreased steadily across urgency levels. ICCs revealed low between-center variability, which did not decrease after accounting for U-scores.

**Conclusions:**

Higher NTS urgency classifications are associated with increased referral rates during fever-related out-of-hours GP consultations. However, overall referral rates remain low, while a substantial number of consultations are pre-classified by the NTS as low urgency, contributing to a high workload of unnecessary consultations that highlights the need for more efficient triage and resource allocation.

Key messages

- Febrile children contribute to heavy workload in out-of-hours GP cooperatives.
- Only 11.6 (U2) and 3.7% (U3) of febrile children are referred to secondary care.
- 24.5% of fever consultations are low urgency (U4–5); 1.4% are still referred.
- Referrals drop from 29.1% to 0.8% as the urgency level decreases (U1-5).
- Urgency levels U3 & U4 have the highest rates of antibiotic prescriptions.

## Background

In The Netherlands, out-of-hours care is organized in large General Practice (GP) cooperatives, with 50 to 200 General Practitioners (GPs) working in rotating shifts [1, 2]. Triage nurses receive calls, assess the urgency of patients' complaints, and decide whether the patients require attendance, by whom, within what time frame, and the type of help (e.g. if there is need for an ambulance). The Dutch Triage Standard (NTS) is a tool used in out-of-hours care in The Netherlands to help triage nurses, often faced with limited and incomplete information, assess the urgency of medical situations [2].

Based on a series of questions, the NTS classifies the urgency of patients into 6 U-scores and determines how fast a patient needs to be seen by a medical professional. These six levels are defined as: U0 and U1 require immediate hospital action; U2 and U3 are directed to out-of-hours GP care within the next few hours, and U4 and U5 are asked to go to the GP during regular office hours or are given telephonic advice [3]. This score can be overruled by the nurse with or without consulting the supervising GP due to logistical constraints, such as a busy schedule at the care center, or a nurse's instincts that lead to either escalation or de-escalation of the urgency level [3, 4].

Available literature regarding the effectiveness of the NTS in The Netherlands has shown conflicting results. Some have shown a clear association between U-scores and resource allocation, hospital admissions, and follow-up visits [5], supporting its ability to properly identify and classify the urgency of patients' needs [6]. Other studies have shown that there is room and need for improvement in the decision accuracy of the triage system [2, 7, 8].

In cases of febrile children, the NTS system relies on a caller's ability to describe symptoms [9]. Assessing a child's condition remotely is difficult, as it relies on the child's appearance, unclear symptoms, and is influenced by parental anxiety and emotional distress [10]. Parents may insist on an immediate response, motivated by fear or uncertainty, even in non-urgent situations, influencing the decision-making of triage nurses and leading to a potential overestimation or underestimation of the level of urgency [11].

Fever-related consultations account for a substantial part of out-of-hours GP workloads, particularly among children under the age of 12 [2, 11, 12]. However, many of these consultations could likely have been managed with lower levels of urgency if classified properly [10]. This highlights the importance of evaluating the efficiency of the NTS in distinguishing between serious and unserious fever related illnesses in children. Failing to do so risks both overburdening healthcare resources with non-urgent cases and exacerbating illness in seriously febrile children due to delayed or inappropriate care.

## Objectives

The aim of the current study is to investigate the agreement between pre-consultation NTS urgency classifications (U-scores) and post-consultation management decisions in febrile children and examine whether the NTS helps reduce variation in decision-making across out-of-hours GP cooperatives.

## Methods

### Study design and population

This secondary analysis uses data from the CHILI study, a cluster-randomized trial conducted by de Bont et al. between 2015 and 2016 across 20 out-of-hours GP cooperatives using the Call Manager software (Labelsoft Clinical IT B.V., CompuGroup Medical). In the CHILI study, the effectiveness of an interactive illness-focused booklet was evaluated by comparing antibiotic prescribing and related outcomes between out-of-hours GP centers with access to the booklet and those providing usual care. The intervention was ineffective because access alone did not alter prescribing behavior. Twelve percent of children in the study population were seen by a family physician who used the booklet, and in this group antibiotics prescriptions were slightly lower compared to usual care (25% versus 22%)[13]. The full CHILI study population was used as the sample for this analysis [13]. Patients selected for the study consisted of all consultations for children between the ages of 3 months and 12 years of age that were identified to be fever related by the our-of-hours GP [13]. The original CHILI study included 31 648 fever-related out-of-hours GP consultations without applying exclusion criteria.

### Outcomes

The primary outcome of this study is immediate referral to secondary care (e.g. emergency department, ambulance services, or pediatricians) during an out-of-hours GP consultation. The secondary outcomes were antibiotic prescription and prescription of other medications, such as nasal sprays and inhalers, during out-of-hours GP consultations. These three outcomes were used as proxies of the actual seriousness of a fever-related illness for which a consultation at an out-of-hours GP cooperative was made. The data on these variables was recorded by the GP after each consultation, then collected from the electronic health records and provided to the researchers in the CHILI study by an independent party, Labelsoft Clinical IT B.V. () Because referral and the two prescription outcomes could co-occur in one consultation, the number of consultations without any of these three outcomes cannot be inferred from descriptive statistics on the individual outcome variables. We therefore explicitly quantified consultations in which none of these outcomes occurred and examined their association with U-scores.

### Independent variables

The main exposure variable was the urgency level (U-score) determined by the triage nurse using NTS before the out-of-hours GP consultation. Furthermore, models were adjusted for overruling of the U-score by the triage nurses and patient (gender and age) characteristics, as they can be possible confounders. Models were also adjusted for use of the intervention booklet (measured by a pop-up question after the consultation during the CHILI study), as this might be directly influencing the outcomes (particularly antibiotic prescriptions). All data were collected from the same electronic health records as the outcome variables.

### Statistical analysis

We performed a series of descriptive statistics to characterize the study population, using frequencies and percentages for categorical variables and medians and interquartile ranges for continuous variables (age).

The main analysis comprised three parts. First, the distribution of U-scores and outcome rates, including referrals to secondary care, antibiotic prescriptions, and other medication use, was illustrated in bar graphs to present the frequency of each triage classification and subsequent outcomes. Second, logistic regression models incorporating natural cubic splines and a random intercept for out-of-hour GP cooperative were created to evaluate the adjusted association between U-scores and each outcome. Natural cubic splines with three and five knots were used for urgency level (33.6th and 66.7th percentiles) and age (20th, 40th, 60th, and 80th percentiles) [14]. Adjusted probabilities derived from these models were visualized in line graphs to facilitate interpretation, and Log-likelihood ratios were used to compare the model fit between models with and without the U-scores and overruling of the U-scores. Third, intraclass correlation coefficients (calculated as: σ^2^_u_/(σ^2^_u_ + π^2^/3)) were used to explore the between-center variance in outcomes before and after adjusting for U-scores, allowing for the assessment of the extent to which the NTS standardized clinical decision-making across out-of-hour GP cooperatives.

A prespecified sensitivity analysis assessed the impact of nurse overruling of the U-scores by including interaction terms in the models, between the U-scores and whether they were overruled. In a post-hoc analysis, the association between the U-scores and no outcome was explored, using the same modeling approach described above. Then, an analysis was conducted on a restricted sample that excluded those consultations referred to secondary care to examine the distribution of antibiotics and other prescriptions. Finally, the sample was stratified according to the type of triage performed, categorized by primary complaints (sick child, febrile child, cough, and shortness of breath), to explore how different types of triages influence the effectiveness of the NTS.

A complete case analysis was carried out. Approximately 5% of the consultations contained missing values, only in variables related to triage U-scores and the use of the intervention booklet. A substantial proportion of these missing values occurred in cases such as reconsultations, ambulance advice requests, and walk-in consultations. These types of encounters were justifiably excluded from the analysis because they were not aligned with the study objective, which focused on the triage and management of febrile children during out-of-hours GP consultations. As described by Bartlett et. al. in 2015 the regression coefficients of the U-scores from a complete case logistic regression analysis will be asymptotically unbiased unless the missingness in U-scores or booklet use are jointly dependent on U-score itself and the outcome (i.e. referral, antibiotics, other prescription), and possibly confounders [15].

The power calculations are shown in the supplementary analysis Section 6. All analyses were conducted in R using the *rms* package for regression modeling and *lme4* for multilevel models. Results were reported with 95% confidence intervals and *P*-values to three decimal places. Formal significance testing was limited to the interaction analysis and used a significance threshold of α = 0.05.

## Results

**Figure 1:**
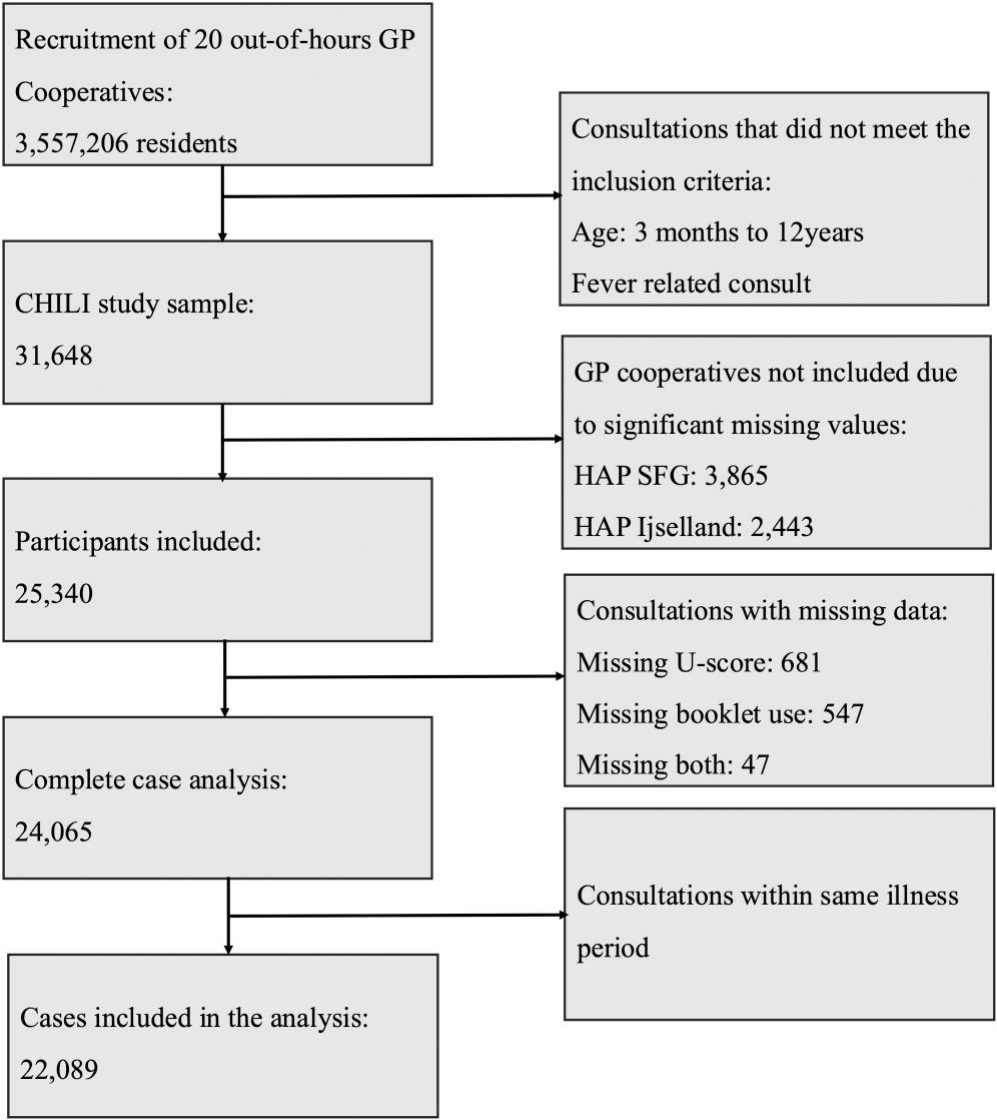
Flow chart of the recruitment, inclusion, and exclusion of the study population.

A total of 31 648 fever related consultations were identified among the 20 GP out-of-hours cooperatives from November 2015 through May 2016. The cooperatives HAP SFG Rotterdam Noord and HAP IJsselland were excluded from the analysis due to high rates of missing values, which reflect poor data quality in their triage documentation. Furthermore, only index consultations with complete data on all variables were selected. After applying these exclusion criteria, 22 089 patients were available for the present analysis (Fig. 1). In total, 6.1% were referred to secondary care, 19.5% were prescribed antibiotics, 10.9% were prescribed other medications, and 64.3% had no outcome. Additional characteristics of the children included in the analysis and their consultations at the out-of-hours GP cooperatives are presented in Table 1.

**Table 1:** Characteristics of the 22 089 out-of-hours GP consultations for febrile children under the age of 12 in The Netherlands between 2015 and 2016 included in the final study sample after the inclusion and exclusion criteria were applied.

Sample characteristics	Study sample (n = 22 089)
Referred to secondary care (%)	1347 (6.1%)
Antibiotic prescription (%)	4316 (19.5%)
Other medications (%)	2417 (10.9%)
No outcome (%)	14 215 (64.4%)
Age at consult, median (IQR)	2 (1–4)
Sex Male (%)	11 608 (52.6%)
Sex Female (%)	10 481 (47.4%)
Use of Booklet (%)	3100 (14%)
U-scores overruled (%)	1722 (7.8%)
U-score 1	265 (1.2%)
U-score 2	3791 (17.2%)
U-score 3	12 615 (57.1%)
U-score 4	1937 (8.8%)
U-score 5	3481 (15.8%)

**Figure 2:**
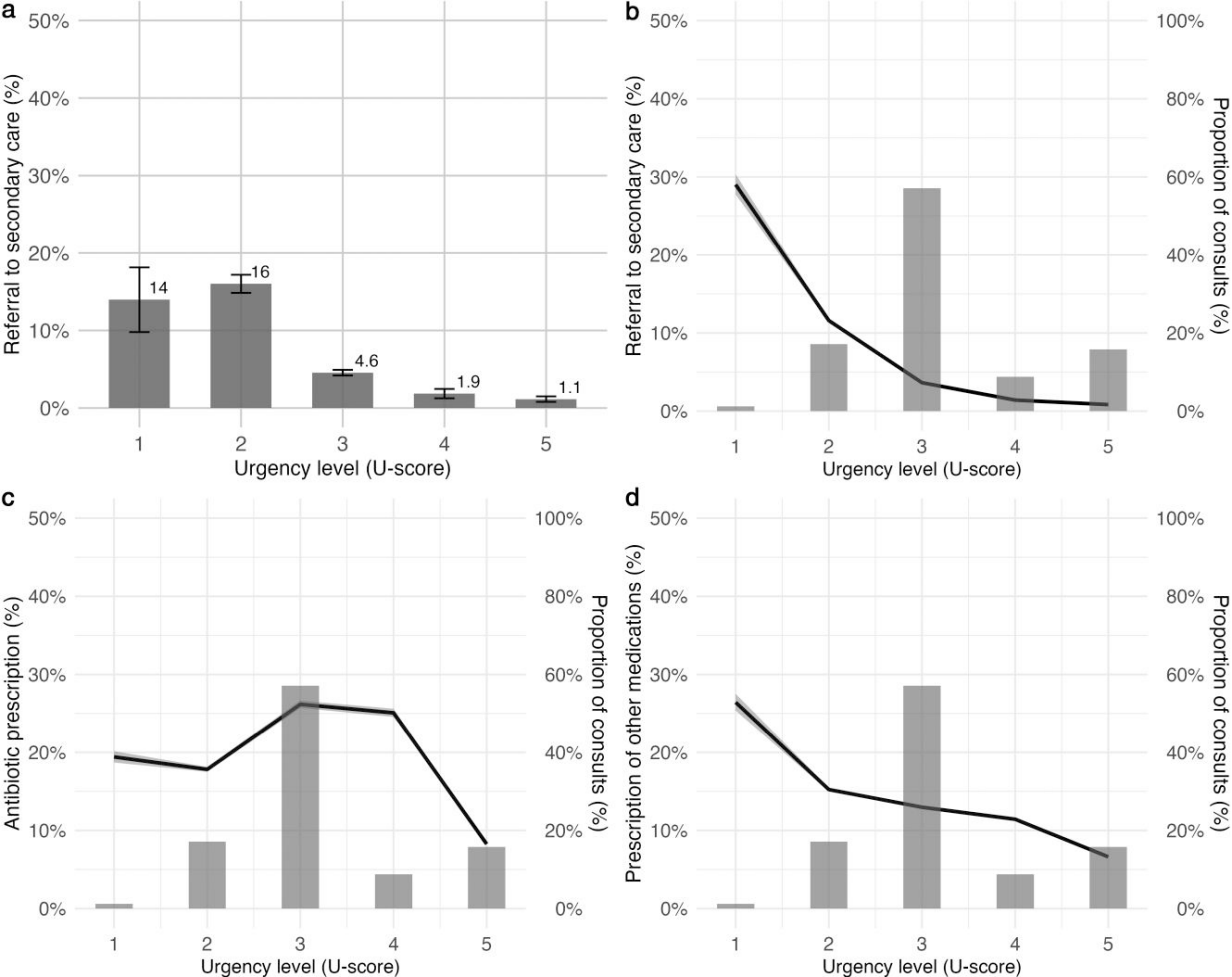
Percentages of referral to secondary care, antibiotic prescription, and prescription of other medications across urgency levels (U-scores). Panel a. shows the unadjusted raw percentages of referrals to secondary care across U-scores. Panel b. presents the adjusted percentages of referrals to secondary care after controlling for patient characteristics (age and gender) and case characteristics (U-score overruling and use of the informative booklet), using a natural cubic spline for urgency level and age. Panels c. and d. show the adjusted percentages of antibiotic prescriptions and prescriptions of other medications, respectively, also adjusted for the same patient and case characteristics. The adjusted percentages were estimated by setting covariates to fixed reference values: age of 2.71 years, male sex, no U-score overruling, and no use of the booklet. In Panels b., c., and d., the black line represents the predicted outcome, the shaded area shows the 95% confidence interval, and the blue bars (with values on the right y-axis) indicate the distribution of consultations across U-scores.

The unadjusted percentages of referrals to secondary care among febrile children during out-of-hours GP consultations decrease with decreasing urgency levels (i.e. higher U-scores), going from 33.6% for U-score 1 to 1.1% for U-score 5 (Fig. 2, Panel a). The association was also present after adjusting for patient and case characteristics (χ^2^ = 912.1, df = 4, *P* < 0.001), and the adjusted percentages display a similar downward trend to the raw percentages (Fig. 2, Panel b).

The association between U-scores and antibiotic prescription appears less consistent than that with referrals to secondary care. In the adjusted model, the highest antibiotic prescription rates were observed at U-scores 3 and 4, with 25.9% and 24.8%, respectively (Fig. 2, Panel c). In contrast, the association between U-score and the prescription of other medications closely mirrors the pattern seen for referrals, although the association is less pronounced (Fig. 2, Panel d). Overall, rates of prescriptions of other medications ranged from 26.4% for U-score 1 to 6.6% for U-score 5.

**Figure 3:**
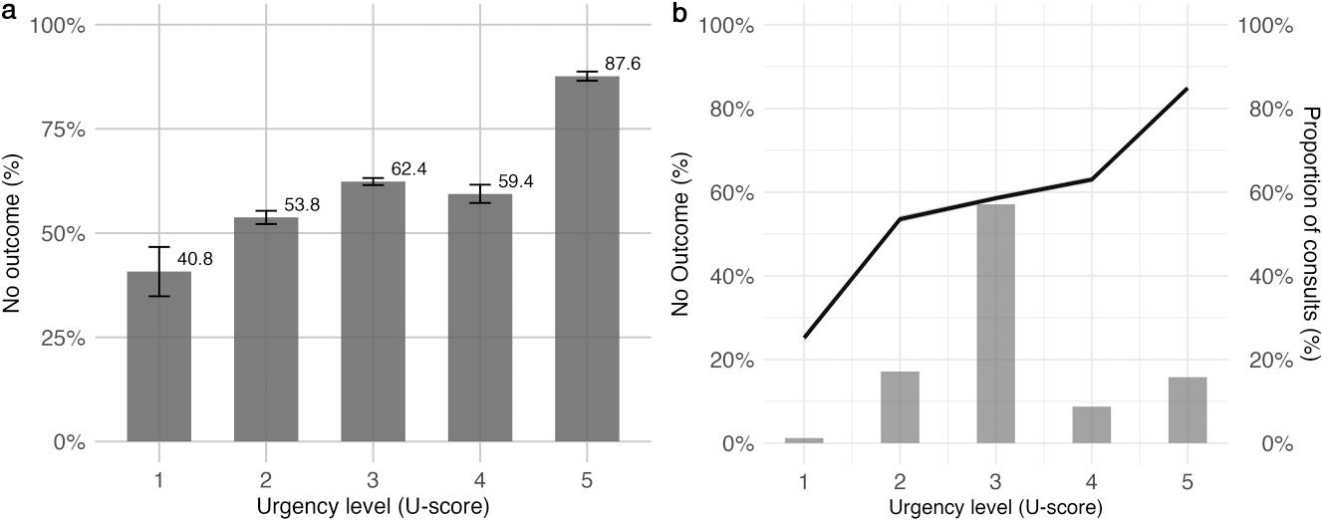
Percentages of out-of-hours GP consultations for febrile children under 12 with no outcome across urgency levels determined by the NTS. No outcome is defined by the absence of referral to secondary care, antibiotic prescription, and prescription of other medication. Panel a presents raw percentages of consultations with no outcome for each U-score. In panel b, the black line shows adjusted no outcome percentages for a patient with covariates set to fixed reference values: age of 2 years 9 months, male sex, no U-score overruling, and no use of the booklet. The black line represents the adjusted outcome, the shaded area shows the 95% confidence interval, and the blue bars (with values on the right y-axis) indicate the distribution of consultations across U-scores.

Both antibiotic and other medication prescriptions were significantly associated with U-scores (χ^2^ = 547.8, df = 4, *P* < 0.001; and χ^2^ = 170.1, df = 4, *P* < 0.001, respectively). Similar patterns were observed in both unadjusted and adjusted models, as shown in Supplementary Figure A2. Full model specifications and the corresponding odds ratios are presented in supplementary Analysis Section 1. Furthermore, an analysis of the association between the U-scores and no outcome during the out-of-hours GP consultations showed that no outcome increased almost linearly with the decrease in urgency level (increase in U-score) (Fig. 3).

The intraclass correlation coefficients (ICCs), reflecting the proportion of the total variance in the outcomes that is attributable to between-center variation across out-of-hours GP cooperatives, were low for all outcomes and showed only minimal reduction after adjustment for NTS U-scores. The ICCs for referral to secondary care, antibiotic prescription, and prescription of other medication were 1.64%, 1.75%, and 1.03%, respectively, and declined only minimally (≤0.3%) after adjustment for U-scores, indicating low between-center variation.

A total of 1722 (7.8%) triage U-scores were overruled. The interaction between urgency levels (U-score) and the overruling of these scores by the triage nurses did not have a significant effect on the referral to secondary care (χ^2^ = 2.253, df = 3, *P* = 0.522). Nevertheless, the interaction was significant for both antibiotic prescription (χ^2^ = 8.52, df = 3, *P* = 0.036) and prescription of other medication (χ^2^ = 17.52, df = 3, *P* < 0.001) (Supplementary Table A2). A simple effects analysis indicated that despite the statistical significance of these interactions, the outcome percentages across urgency levels were similar for cases overruled and cases not overruled (Supplementary Figure A3), thus suggesting limited clinical relevance.

Furthermore, the sensitivity analysis indicated that excluding consultations resulting in referral to secondary care had minimal impact on the observed associations between U-scores and both antibiotic prescription and prescription of other medications (Supplementary Figure A4). This suggests that the association between urgency level and prescribing behaviors remains consistent, even when more severe cases were removed from the analysis. Additionally, stratified analyses by the type of triage performed before the consultation, specifically for the categories Sick Child, Febrile Child, Cough, and Shortness of Breath, indicated that the overall trend in referral practices was consistent across different presenting complaints (Supplementary Figure A5 and A6).

## Discussion

Our findings show that urgency levels assigned during triage were associated with clinical management outcomes during out-of-hours GP consultations for febrile children: the lower the U-score (i.e. the higher the urgency), the higher the GPs' referral rates. Consultations under U-scores 2 and 3 (those typically managed during out-of-hours care) had relatively low rates of referral to secondary care (11.6% and 3.7% respectively), and over half of these consultations showed no recorded clinical outcome, suggesting GPs did not consider the illness to be serious. Interestingly, 24.5% of consultations had U-scores of 4 and 5, despite the NTS recommending these to be managed during regular office hours or via telephone advice. While this may contribute to a higher workload in out-of-hours GP cooperatives, 1.4% of these cases were referred to secondary care, potentially highlighting serious illness missed by the NTS.

When situating our findings in the broader literature, it is notable that van Ierland et al. observed no association between telephone triage urgency and GP decision-making in childhood consultations (though not limited to fever) [16], highlighting that the impact of triage systems on clinical actions may vary by context and condition. Nevertheless, our results align with the conclusions by Smits et. al. (2020), who found that the NTS generally performs reliably and accurately in triaging children. Moreover, the phenomenon of serious cases being missed by the NTS is documented across different outcomes, such as chest discomfort (<18) [7] and shortness of breath in adults [2].

We can observe lower-than-expected referral rates for consultations under urgency score 1, which is counterintuitive since the definition of a U1 score is immediate action. However, due to the extremely urgent nature of these cases, decision-making often bypasses GP consultations or occurs outside documented pathways, resulting in data blind spots. The sample of patients who attend out-of-hours GP care despite a U1 classification therefore represents a selective subgroup of all U1 cases and should be interpreted with caution. Antibiotic prescription rates were relatively higher among lower urgency levels, specifically U-scores 3 and 4 (25.9% and 24.8%, respectively). The higher levels of antibiotic prescription rates observed at these urgency levels may reflect the influence that the presence of a face-to-face consultation has on inadvertently raising parental expectations and contributing to “better safe than sorry” practices by the GP. This dynamic can lead to a perceived need to prescribe antibiotics, even when clinical guidelines advise against their use. Notably, the temporal progression of the illness likely contributes to higher prescribing in moderate urgency cases; children presenting without acute alarming symptoms but with prolonged illness duration may prompt clinicians to prescribe antibiotics as a precaution against potentially missed serious infections. These observations align with findings by Elshout et al. (2012), who reported that only 19% of antibiotic prescriptions are directly attributable to alarm signs and symptoms [9]. Instead, external factors such as parental pressure, prolonged illness duration, and the very occurrence of a face-to-face consultation appear to play a significant role in influencing these decisions [9]. Finally, the clinical significance of consultations assigned to the lowest urgency levels requires careful consideration. While appropriately triaging and redirecting such cases to regular office hours or managing them through telephone advice may contribute to reducing the burden on out-of-hours GP cooperatives, caution is needed because 1.4% of these low-urgency cases (U4-5) nonetheless resulted in referrals to secondary care. Thus, efforts to reduce workload must be accompanied by further research into the clinical significance of these referrals in low-urgency cases and by potential refinement of the NTS to ensure that truly urgent cases are accurately identified.

The between-center variance reflected by the ICC was very low to start with and decreased very little when adjusting for the U-scores. This suggests that the specific out-of-hours GP cooperatives where the consultation took place had minimal influence on clinical management decisions, with most of the variation attributable to differences between individual patients. This minimal reduction might have been caused by the inefficiency of the NTS in standardizing triage processes across out-of-hours GP cooperatives or simply by the fact that there is already very little variance to begin with, and thus there was minimal room for improvement. Additionally, as the data were not collected before and after the introduction of the NTS, the ICC values should be interpreted with caution, as they do not capture changes attributable to the system's implementation.

### Limitations

This analysis utilized data originally collected for a different research purpose, thereby limiting control over the quality, consistency, and validity of the recorded measurements. The key outcomes in this study, referral to secondary care, antibiotic prescription, and prescription of other medications, were used as proxies for clinical seriousness of an illness, a construct for which no objective gold standard exists. Consequently, these outcomes may not fully capture the underlying true severity of illness. Likewise, the absence of any of these three outcomes does not exclude the possibility that the child was seriously ill. Due to the cross-sectional nature of the data, no follow-up information was available to assess the longer-term appropriateness of the clinical decisions made, limiting our ability to evaluate the true accuracy or consequences of GP decision-making. The absence of follow-up data limits our understanding of whether referrals among low-urgency (U4-U5) consultations reflect triage misclassification, appropriate clinical escalation, or unnecessary referrals by the GP. In addition, in a minority of cases, nurses overruled U-scores, but data on the nature of or reasons for overruling were not available. This prevented an in-depth investigation of potential areas of improvement of the NTS.

## Conclusion

This study demonstrates a positive association between urgency levels assigned by the Dutch Triage System (NTS) and referrals to secondary care during out-of-hours GP consultations for febrile children. While the NTS effectively stratifies risk in most cases, the persistence of serious cases being missed highlights important gaps in its sensitivity and clinical application. Further research is needed to better understand how the NTS functions, including the accuracy of U-scores and the role of triage nurses, to inform targeted improvements in its performance. Moreover, the landscape of out-of-hours primary care has undergone substantial changes in recent years, particularly in the aftermath of the COVID-19 pandemic and the incorporation of modern technologies in triage, such as video triage [6]. Updated research is needed to investigate if these patterns of urgency misclassification, as well as the association between the U-scores and referral to secondary care rates, antibiotic prescription, and prescription of other medications are still present.

## Supplementary Material

cmag015_Supplementary_Data

## Data Availability

Data are available on reasonable request by contacting the corresponding author.
