# Supplementary material for: Performance of the Dutch Triage standard in managing fever in children in out-of-hours primary care: a secondary analysis of the chili study
Source: Fam Pract. 2026 Apr 7;43(3):cmag015. doi: 10.1093/fampra/cmag015 (PMC13064533; doi:10.1093/fampra/cmag015)
Supplement: cmag015_Supplementary_Data [file cmag015_supplementary_data.pdf]

Supplementary analysis

Section 1 – Coefficients And Natural Cubic Splines for The Logistic Regression Models

Table A 1. Logistic regression models used to estimate adjusted probabilities of referral to secondary care, antibiotic prescription and prescription of other medications in out-of-hours GP consultations for febrile children. The table presents the fixed effects from 3 multivariable logistic regression models, including Odds Ratios, standard errors and 95% confidence intervals. The table excludes the variables for U-scores and age, since they were modelled using natural cubic splines, the spline graphs for age is shown in Fig. A1, and for U-scores in Fig. 2.

	Model 1: Referral to secondary care		*Model 2: Antibiotic prescription*		*Model 3: Prescription of other medications*	
**Variable**	OR	95% CI	OR	95% CI	OR	95% CI
**Gender**	0.96	[0.86, 1.08]	0.97	[0.91, 1.04]	0.87	[0.79, 0.94]
**Overruling**	1.23	[1.02, 1.49]	1.02	[0.90, 1.16]	1.11	[0.95, 1.30]
**Booklet use**	0.68	[0.57, 0.82]	1.19	[1.07, 1.32]	0.98	[0.86, 1.12]

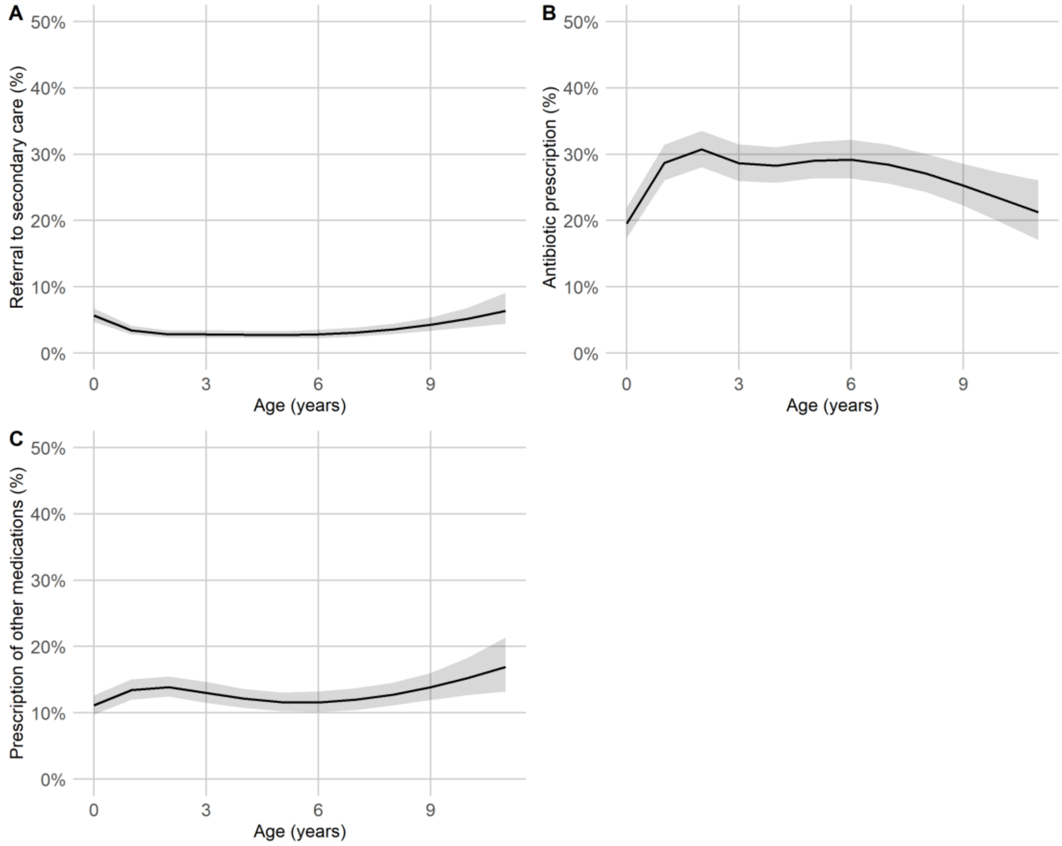

Figure A 1. Natural cubic splines for age, when controlling for all other variables in the logistic regression models.

Section 2: Raw Percentages of U-Scores for Antibiotic Prescription and Prescription of Other Medications

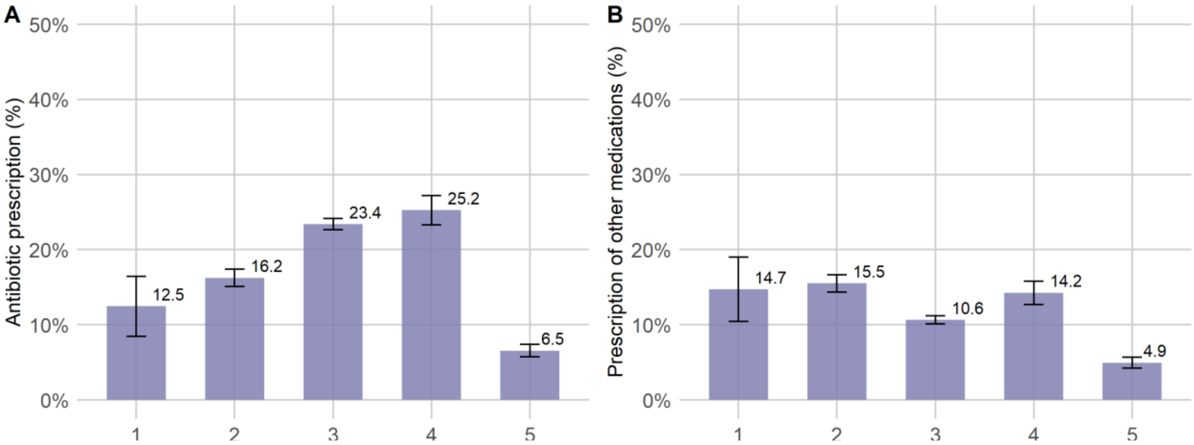

Figure A 2. Unadjusted percentages of antibiotic prescriptions and prescriptions of other medications after out-of-hours GP consultations for febrile children for each U-score in the dataset.

Section 4: Simple Effects Analysis of The Significant Interactions

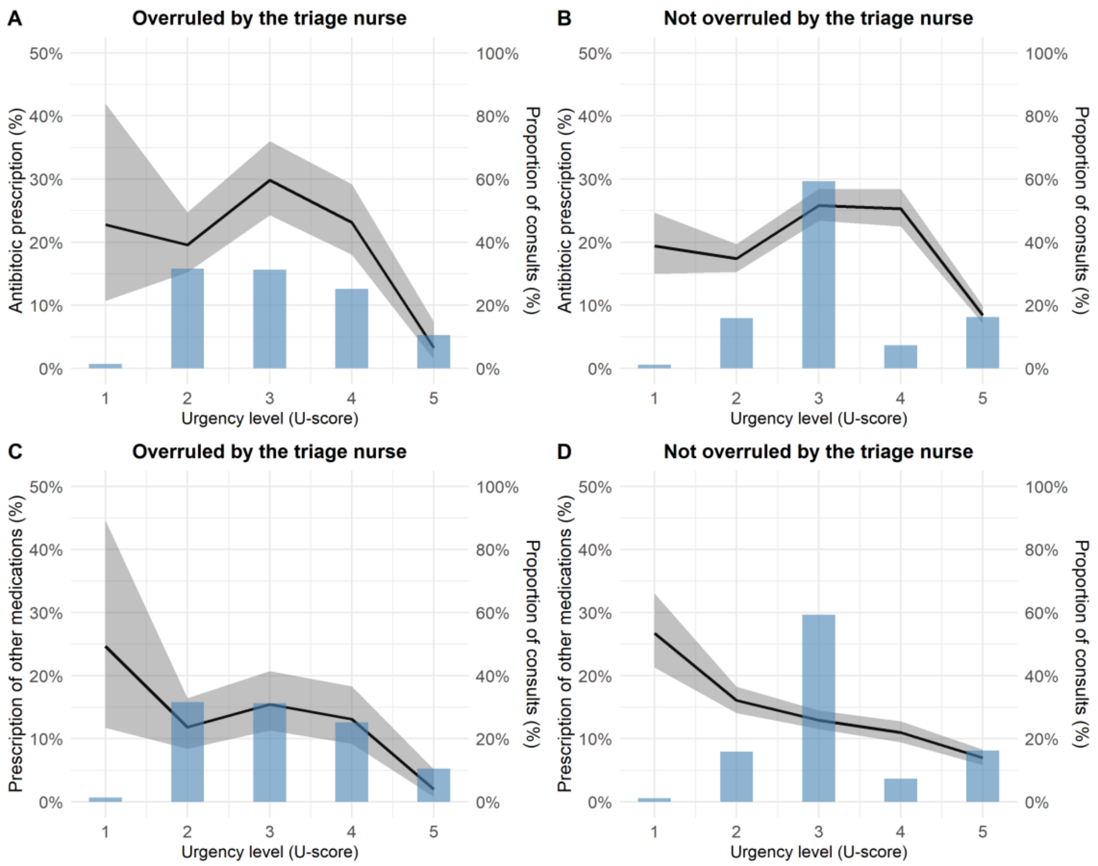

Figure A 3. Simple effects analysis of the prescription of antibiotics and other medications stratified by overruling or not of the initial U-score provided by the NTS by the triage nurse before the out-of-hours GP consultation. Panel a. and c. show the adjusted percentages of antibiotic prescription and prescription of other medications in cases where the nurse did overrule the U-score, and panels b. and d. when there was no overruling.

Section 4: Sensitivity Analysis Excluding Cases with Referral to Secondary

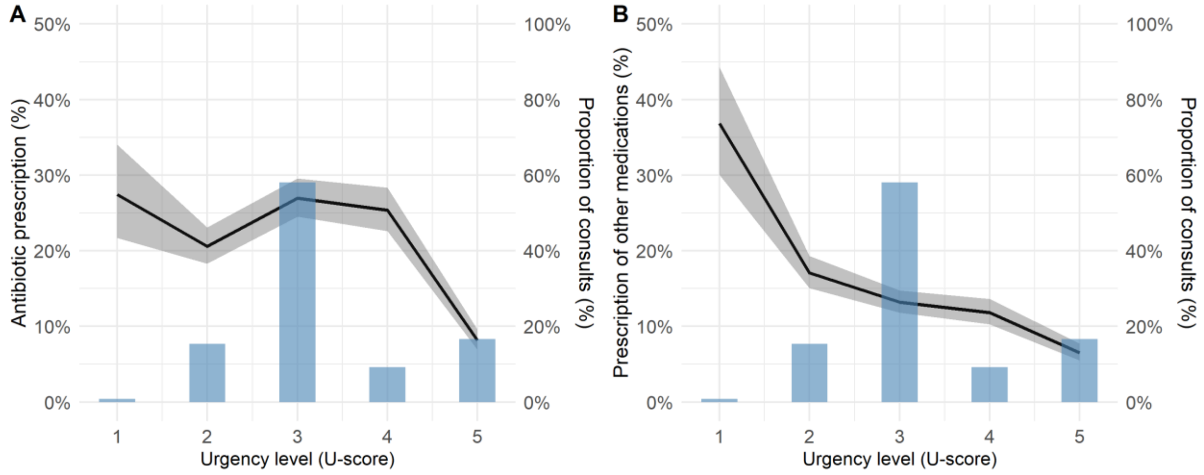

Figure A 4, Percentage of antibiotic prescriptions and prescriptions of other medications by urgency level (U-score) in a sample of out-of-hours GP consultations excluding those referred to secondary care. Values represent the proportion of antibiotic prescriptions and prescription or other medications per urgency level after adjusting for patient characteristics (age and gender) and case characteristics (Overruling of U-scores and use of the informative booklet).

Care

Section 5: Sensitivity Analysis Stratifying by Type of Triage

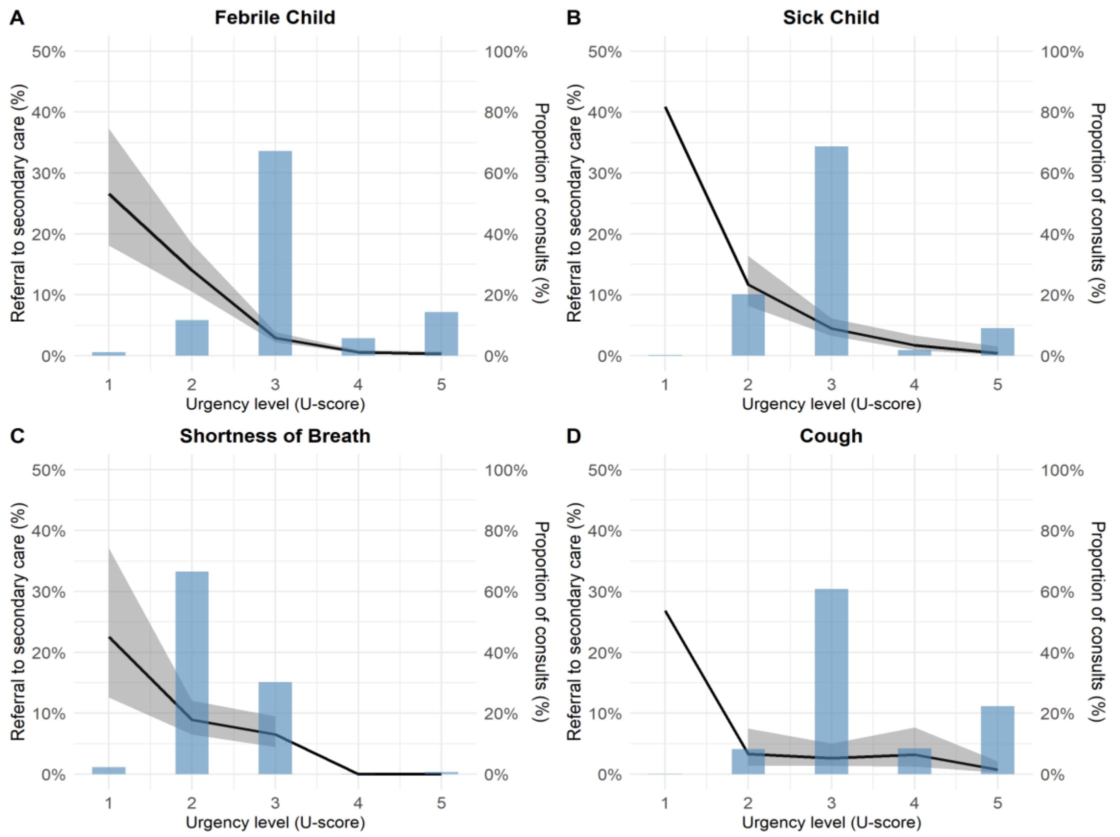

Figure A 5. Percentage of referrals to secondary care across urgency levels (U-score) in a sample stratified by the main presenting symptoms and type of triage carried out to determine the urgency level. Values represent the proportion of referrals to secondary care per urgency level after adjusting for patient characteristics (age and gender) and case characteristics (Overruling of U-scores and use of the informative booklet).

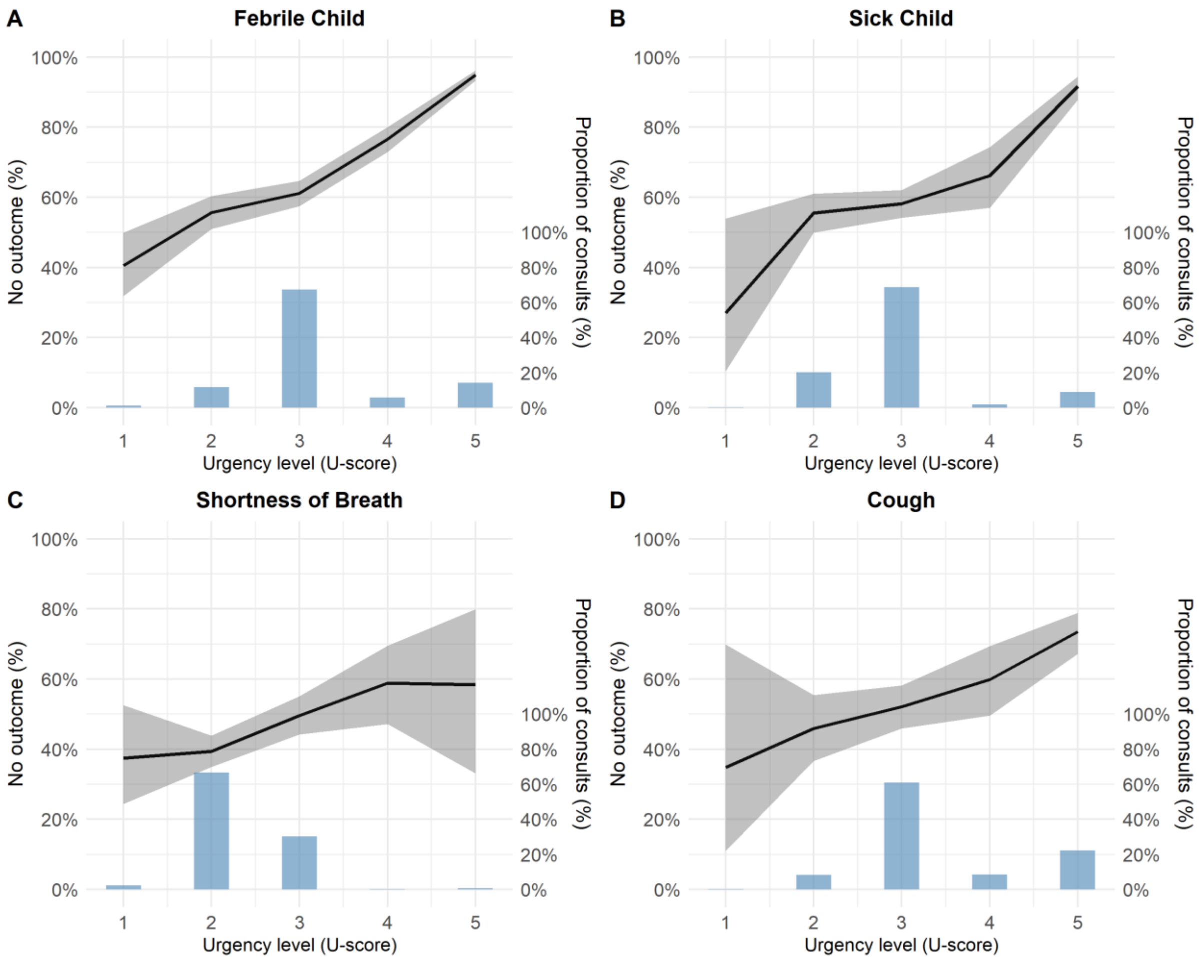

Figure A 6. Percentage of consultations with no outcome across urgency levels (U-score) in a sample stratified by the main presenting symptoms and type of triage carried out to determine the urgency level. Values represent the proportion of consultations with no outcome per urgency level after adjusting for patient characteristics (age and gender) and case characteristics (Overruling of U-scores and use of the informative booklet).

Section 6: Power Analysis

For the purpose of this power analysis, the hypothesis is based on the change in outcome rates between U2 and U3 scores, as these scores are the most relevant for the study and incorporate most of the dataset. The minimal detectable change was calculated assuming a logistic regression model for the main outcome in this study (referral to secondary care). In order to do this, a z-test for logistic regression was used, using the "pwr" package in RStudio. Since the data is clustered a design effect (DE) was applied to calculate the effective sample sizes (ESS) using the following formulas:

$$DE = 1 + (n - 1)\,\rho.$$

$$ESS = (n * k) / DE$$

Where; n = average cluster size, p = ICC of the outcome, and k = number of clusters (18[.

Furthermore, the clusters have different sizes in the dataset, thus the minimal detectable change was calculated with an adjusted sample size for two different scenarios, one for the smallest cluster and one for the average cluster size.

The minimal detectable change in referral to secondary care rates, when the smallest cluster size is considered, is an odds ratio of 1.82, assuming a statistical power of 0.80 and applying a significance level of 0.05 (chosen as conventional thresholds in epidemiological studies). This threshold was determined using an outcome probability (referral to secondary care for febrile children in out-of-hours GP consultations) under the null hypothesis of 0.079. [14] Furthermore, an effective sample size of 302 was used, determined using the smallest cluster size of 264 and an ICC of 0.056. [19]

Using the same assumptions, with an effective sample size of 317.1, determined from an average cluster size of 1,227 the minimal detectable change in referral to secondary care rates is 1.79.
